# Colicins and Salmocins – New Classes of Plant-Made Non-antibiotic Food Antibacterials

**DOI:** 10.3389/fpls.2019.00437

**Published:** 2019-04-09

**Authors:** Simone Hahn-Löbmann, Anett Stephan, Steve Schulz, Tobias Schneider, Anton Shaverskyi, Daniel Tusé, Anatoli Giritch, Yuri Gleba

**Affiliations:** ^1^NOMAD Bioscience GmbH, Halle, Germany; ^2^DT/Consulting Group, Sacramento, CA, United States

**Keywords:** antimicrobials, bacteriocin, colicin, salmocin, plant-based expression system, GRAS, food safety

## Abstract

Recently, several plant-made recombinant proteins received favorable regulatory review as food antibacterials in the United States through the Generally Recognized As Safe (GRAS) regulatory procedure, and applications for others are pending. These food antimicrobials, along with approved biopharmaceuticals and vaccines, represent new classes of products manufactured in green plants as production hosts. We present results of new research and development and summarize regulatory, economic and business aspects of the antibacterial proteins colicins and salmocins as new food processing aids.

## Introduction

Since the early and mid-twentieth century, antibiotics have been amongst the most impactful pharmaceuticals for maintaining public health. However, the broad and indiscriminate use of these medicines has caused evolution of multi-drug resistant (MDR) bacteria that are increasingly insensitive to multiple antibiotic classes, including so-called antibiotics of last resort, such as carbapenems, colistin, and third- and fourth-generation cephalosporins. The threat of MDR pathogens is fully recognized by the World Health Organisation (WHO) and governments worldwide, but coherent actions for integrated management of MDR pathogens are still lacking (Tacconelli et al., 2018). Most antibiotics on the market today are generic drugs and are inexpensive, and pharmaceutical companies have few incentives to develop new classes of antimicrobials. The pipeline of antimicrobials currently in clinical trials includes predominantly modifications of the earlier discovered classes and do not offer novel modes of action; thus, it is fully expected that the pathogens will evolve to become resistant. The pathogens most difficult to control are Gram-negative bacteria such as *Campylobacter, Pseudomonas, Escherichia*, and *Salmonella*, as those pathogens have developed resistance to most or all existing antibiotic classes. Novel non-antibiotic antibacterials are one approach to solving the MDR problem and are thus urgently needed.

Many major health threats including the Gram-negative bacteria *Salmonella enterica, Escherichia coli*, and *Campylobacter jejuni*, and the Gram-positive bacteria *Listeria monocytogenes* and *Clostridium perfringens* are food-borne pathogens. Food-related bacterial outbreaks are occurring with increasing frequency and severity. The problem is exacerbated by the globalization of food manufacturing processes whereby the food is produced and transported from different continents and mixed or blended before use, thus amplifying potential pathogen spread. For example, a hamburger bought at a fast-food restaurant normally contains meat from over 100 different animals, meaning that meat from one infected animal may infect hundreds of customers^1^. Driven by customer demands for so-called ‘organic’ food production practices, many farmers and companies try to reduce the environmental impact of their operations by avoiding the use of chemicals and antibiotics in the process of animal rearing, plant production and food preparation, and instead use traditional methods of husbandry and agriculture, such as use of animal dung as a fertilizer, or keeping animals and plants in close proximity. These practices may introduce additional risk of bacterial contamination not only to domestic animals but also to vegetables grown in nearby fields that may become exposed to contaminated irrigation water and run-off^2^. Bacteria such as *Escherichia, Salmonella*, and *Listeria* are very promiscuous and can survive, and even multiply, in plants, despite the fact that their normal hosts are animals. It is symptomatic that during the last decade, more food-related outbreaks are the result of consumption of infected plants or plant sprouts rather that the animals that are the main reservoir of the pathogenic bacteria^3^.

Several research teams have searched for antibiotic alternatives, and in particular, attempted development of non-antibiotic antibacterial proteins derived from bacteria (*E. coli* colicins and colicin-like molecules) and bacteriophages (endolysins or “lysins”) for control of bacterial pathogens. *Escherichia* colicins and colicin-like molecules derived from other Gram-negative bacteria are surprisingly easily and well-expressed in plants, are fully functional and are up to 10^6^ times more potent than antibiotics on a molar basis (Schneider et al., 2018). Due to their nature, however, these molecules are narrowly specific and typically cocktails of these proteins are needed for good control of all pathovars of the bacterial species. Antibacterial proteins are being developed as new medicines, as antibacterials for food, or both. Nomad Bioscience GmbH (Halle, Germany) and its subsidiary Nomads UAB (Vilnius, Lithuania) are in the forefront of these research efforts, with an early emphasis on the food antimicrobials market. In particular, using the GRAS (Generally Recognized As Safe) regulatory process in the United States, Nomad has already obtained favorable regulatory review and marketing allowance from the United States Food and Drug Administration (FDA) for its *Escherichia*-derived antibacterial proteins, colicins, for use in food. The company has also submitted GRAS notices to FDA for its salmocins, colicin-like proteins derived from *Salmonella*. Similarly, Nomads, Lithuania, has used the GRAS process to confirm marketing allowance of its *Clostridium* phage lysins.

We summarize herein results of new research and development for two classes of antibacterial proteins, colicins and salmocins, that are being developed by Nomad for the food industry as food processing aids. Our discussion includes perspective on key commercialization aspects of these product candidates, including industrial manufacturing in green plants, the quality attributes of these proteins including antibacterial activity *in vitro* and on food matrices, the pathway for regulatory marketing allowance of these products, and the breadth of potential market applications. Current challenges to the commercial adoption of these products are also discussed.

## Materials and Methods

### Bacterial Strains and Growth Conditions

*Escherichia coli* DH10B and STEC as well as *S. enterica* ssp. *enterica* cells were cultivated at 37°C in LB medium [lysogeny broth (Bertani, 1951)]. *L. monocytogenes* cells were cultivated in BHI (Brain heart infusion broth, #X916 purchased from Carl Roth GmbH, Karlsruhe, Germany) medium at 37°C and *Agrobacterium tumefaciens* ICF320 (Bendandi et al., 2010) cells were cultivated at 28°C in LBS medium [modified LB medium containing 1% soya peptone (Duchefa, Haarlem, Netherlands)].

### Plasmid Constructs

Constructs used were described in Schulz et al. (2015) or Schneider et al. (2018).

### Plant Material and Transient or Transgenic Bacteriocin Expression

*Nicotiana benthamiana* WT was grown and transfected with *Agrobacterium* for transient expression as described in Schulz et al. (2015). The generation of bacteriocin-transgenic *N. benthamiana* was published in Schulz et al. (2015) and Schneider et al. (2018). Methods for EtOH-induction of transgenic plants were described in Werner et al. (2011).

### Protein Analysis

Plant leaf material was ground in liquid nitrogen and total soluble protein extracts were prepared with 5 vol. 50 mM HEPES pH 7.0, 10 mM K acetate, 5 mM Mg acetate, 10% (v/v) glycerol, 0.05% (v/v) Tween-20, 300 mM NaCl and the protein concentration of TSP extracts was determined by Bradford assay using Bio-Rad Protein Assay (Bio-Rad Laboratories, GmbH, Munich, Germany) and BSA (Sigma-Aldrich, Co., St. Louis, MO, United States) as a standard if not stated otherwise. Determination of antimicrobial protein concentration in TSP extracts was done semi-quantitatively by comparison of different amounts of TSP extracts with known amounts of BSA on Coomassie-stained SDS/PAGE gels. Protocols for protein purification and purity analysis are described in Stephan et al. (2017) and Schneider et al. (2018).

### Bacteriocin Antimicrobial Activity Determinations

Semi-quantitative and quantitative determinations of antimicrobial bacteriocin activity by a spot-on-lawn soft agar overlay assay or enumeration of viable counts via dilution plating from liquid cultures was done as described in Schulz et al. (2015).

### Reduction of Bacterial Populations on Different Food Matrices

Protocols for *E. coli* contamination of beef trims prior or without grinding and lamb loin with subsequent colicin treatment were similar to Schulz et al. (2015) whereas protocols for contamination of chicken meat, egg, tuna, and beef meat with *S. enterica* and subsequent salmocin treatment were basically described in Schneider et al. (2018).

## Results

### Colicin Biology

Colicins are antimicrobial proteins produced by certain strains of *E. coli* for control of other strains of the same or related species. Colicin genes are carried on colicinogenic plasmids and are part of colicin operons, which include also genes for immunity proteins and lysis proteins. Immunity proteins protect colicin-producing cells against cytotoxic activity of accumulated colicin; the immunity protein gene is expressed constitutively. The lysis protein is expressed as a read-through of colicin gene STOP-codon; being accumulated to critical level, the lysis protein destroys the colicin-producing cell and results in the release of colicin to the environment (Cascales et al., 2007; Kleanthous, 2010; Kim et al., 2014).

**FIGURE 1:**
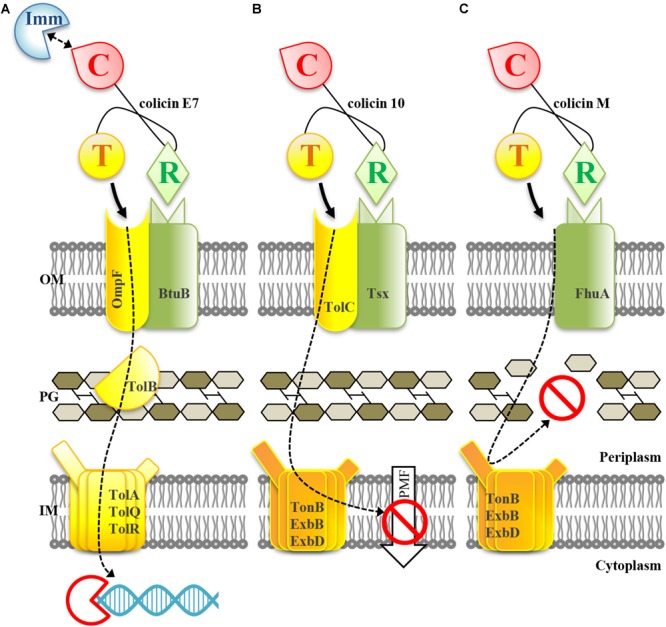
Mechanisms of antimicrobial action of colicins with nuclease **(A)**, pore-forming **(B)**, and murein synthesis inhibiting **(C)** activities. The receptor-binding domain (R) of colicin binds to the outer membrane (OM) receptor of the target cell. The translocation of the colicin molecule into the cell is mediated by interaction of the translocation domain (T) with a cell translocation machinery (Tol or Ton systems). **(A)** Colicins with nuclease activity are translocated across the outer membrane, cell wall and inner membrane (IM) to the cytoplasm where they hydrolase DNA or RNA of the target cell; nucleic acid degradation is catalyzed by the cytotoxic domain (C) of colicin. **(B)** Pore-forming colicins are translocated across the outer membrane and cell wall to the inner membrane. The cytotoxic domain is inserted into the inner membrane and forms a pore which destroys the proton motive force (PMF) and impairs the membrane integrity. **(C)** The murein synthesis inhibiting colicin M is translocated across the outer membrane; it exerts its cytotoxic activity in the periplasm by enzymatic degradation of lipid I and lipid II peptidoglycan intermediates. The cleavage occurs between the lipid moiety and the pyrophosphoryl groups; this results in the arrest of peptidoglycan polymerization. The figure is based on Cascales et al. (2007); Kleanthous (2010), Kim et al. (2014).

Mechanisms of colicin antimicrobial action are summarized in Figure 1. To enter target cells, colicin proteins first bind to outer membrane cell surface receptors (FhuA, OmpF, BtuB, etc.); the translocation across the cell membrane is operated by innate cell translocation machinery (either Tol or Ton transport systems) that is recruited by the colicin translocation domain. Colicins exert three types of cytotoxic activities. Colicins with nuclease (DNase and RNase) activities (e.g., colicins E2-E9) enzymatically degrade DNA or RNA of the target cell (Figure 1A). Pore-forming colicins or porins (e.g., colicin Ia, Ib, K, and U) impair the integrity of cell membranes resulting in cell death due to cell membrane depolarization (Figure 1B). Inhibitors of cell wall biosynthesis exert their bacteriolytic effect via enzymatic degradation of undecaprenyl phosphate-linked peptidoglycan (murein) precursors (Figure 1C). In *E. coli*, this last group is represented only by colicin M. All colicins have a three-domain structure with the N-terminal translocation domain responsible for the transport of the protein across the cell membrane and periplasmic space; the central receptor-binding domain responsible for binding to the outer membrane cell surface receptor; and the C-terminal cytotoxic domain responsible for exerting the killing effect on the target. There is one exception to this convention, namely, the mechanism of translocation of colicin N is not yet clear (Jakes, 2014).

Most pathogenic species of Gram-negative bacteria employ bacteriocins evolutionarily similar to colicins; those are referred to as colicin-like molecules and are given names usually derived from the name of the genus. Apart from *Escherichia* colicins and *Pseudomonas* pyocins, other colicin-like proteins are much less studied (Cascales et al., 2007; Riley, 2009), and the molecular structure and design of some bacteriocins, for example, *Pseudomonas* pyocins, are more diverse (Barreteau et al., 2009; Ghequire and De Mot, 2014; Paškevičius et al., 2017).

There is a growing number of publications dealing with chimaeric bacteriocins engineered to contain domains derived from different proteins and naturally occurring bacteriocins (e.g., colicin Ia; Qiu et al., 2003; Gupta et al., 2013; Behrens et al., 2017); Naturally occurring bacteriocins are synthesized in bacteria that are commensal in the human intestinal tract. As such, they are benign and have not been associated with adverse effects. This feature of natural bacteriocins allows for their treatment as GRAS substances during regulatory review. Hybrid or chimaeric molecules are not discussed here because their record of safety is not yet “generally recognized” and as such they are unlikely to initially qualify for review via the GRAS process.

Bacteriocins are proteins and are thus fundamentally different from commonly used small molecules antibiotics; those differences include much higher molecular size, higher molar activity, limited bioavailability, narrow specificity, and different mechanisms of action. Consequently, they can’t be used as simple replacements for antibiotics and initial indications may be limited to their topical use against known pathogenic species. At the same time, being novel antibacterials, medicinal bacteriocins (e.g., Brown et al., 2012) could command much higher prices in those new indications once they are proven safe and effective (Table 1).

**Table 1:** Bacteriocins versus antibiotics: major biological differences and market potential of bacteriocins.

Antibiotics	Bacteriocins
Size 0.3–0.5 kDa	Size 15–80 kDa
Broadly active	Very narrow, mostly intraspecific selectivity
Effective dose, w/w – 50 mg/kg	Effective dose, w/w – 0.5–5 mg/kg (10–100 times less)
Effective dose, molar basis	Effective dose, molar basis -10^3^–10^6^ lower
Can be used when target pathogen is unknown	Can be used against known pathogens only
Systemic penetration throughout body organs, broadly deliverable	Expected to be efficient for topical applications only (topical, inhalable, ingestible, injectable in blood stream or urogenital tracts)
Mature market ($60 B)	In development as alternatives
Market rapidly eroded because of multi-drug resistance	MDR bacteria are sensitive to bacteriocins
Generic pressure on price	Could command much higher prices as novel individualized medicines in (initially) niche markets
Poor pipeline of new products, low investments by companies in R&D	Research indicates early targets (skin, lung, urogenital tract, gastrointestinal tract, and blood infections)

### Plant-Made Colicins

**FIGURE 2:**
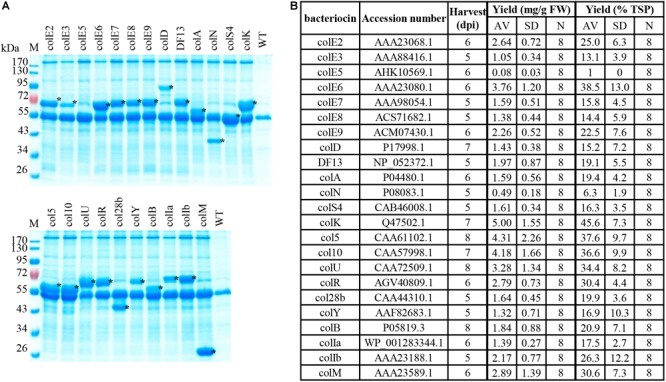
Plant expression of colicins. Transient expression in *N. benthamiana* upon syringe infiltration with 1:100 dilutions of agrobacterial cultures carrying TMV or TMV and PVX vectors. Recombinant proteins were analyzed in TSP (total soluble protein) extracts of leaf tissue prepared with 5 vol. 50 mM HEPES pH 7.0, 10 mM K acetate, 5 mM Mg acetate, 10% (v/v) glycerol, 0.05% (v/v) Tween 20^®^, 300 mM NaCl. **(A)** Coomassie-stained SDS protein gels loaded with TSP extracts prepared from plant material expressing bacteriocins or from (WT) non-transfected leaf tissue; loading corresponds to 1.5 mg FW plant material. Asterisks mark recombinant proteins. **(B)** The yield is given in mg recombinant colicin/g fresh weight of plant leaf biomass and as a percentage of TSP and is represented as an average value and standard deviation (AV, SD) of several experiments. N, number of independent experiments. Transient expression and yield determination were done as described in Schulz et al. (2015). Plant material expressing bacteriocins was harvested at timepoints in days post inoculation (dpi) as indicated in **(B)**.

We selected sequences of 23 (almost all) colicins available in public databases (Figure 2B) and expressed them in *N. benthamiana* plants using the magnICON^®^ system (Gleba et al., 2005, 2014; Klimyuk et al., 2014). Expression of colicins E2, E3, E6, E7, D, N, K, 5, U, B, Ia, and M was described in our previous publication (Schulz et al., 2015). Figure 2A shows the SDS-PAGE analysis of expression for all 23 colicins we tested, including colicins described before. The expression level varied between 0.49 ± 0.18 mg/g FW (6.3 ± 1.9% TSP) for ColN and 5.00 ± 1.55 mg/g FW (45.6 ± 7.3% TSP) for ColK with a majority of colicin proteins expressed at levels between 1 and 3 mg/g FW. ColE1, which was found to accumulate at the lowest yield (approximately 1% of TSP), was excluded from further studies, although it demonstrated antimicrobial activity against some *E. coli* strains (data not shown).

We also successfully expressed some colicins in *Spinacia oleracea* (spinach) plants. The expression levels in spinach, however, were approximately 10-times lower than in *N. benthamiana* (Schulz et al., 2015).

**FIGURE 3:**
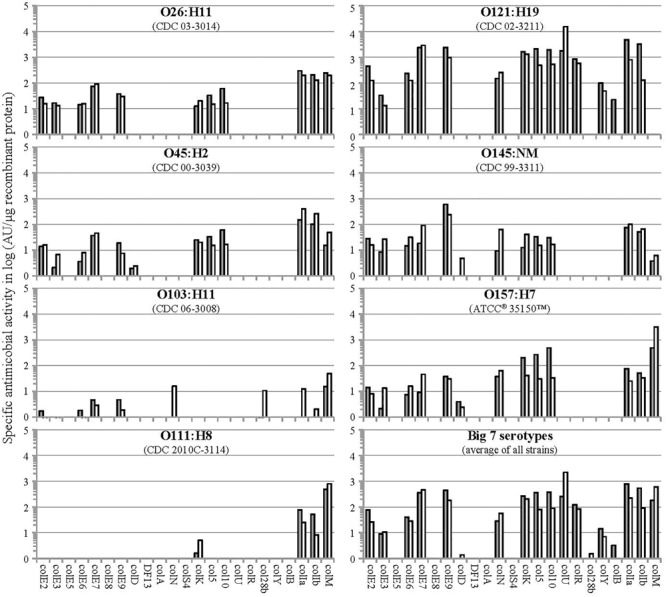
Specific activity of individual colicins against *E. coli* strains of Big7 STEC serovars. Semi-quantitative evaluation of the antimicrobial activity of colicin-containing plant TSP extracts was done by radial diffusion assay via spot-on-lawn-method; the specific antimicrobial activity was calculated in arbitrary units (AU) per μg recombinant protein (gray and white bars correspond to data points of 2 independent experiments).

Antimicrobial activities of plant-made colicins against shiga-toxin producing *E. coli* strains comprising the “Big 7” STEC USDA-FSIS panel^4^ were studied using a spot-on-lawn soft agar overlay assay as described in Schulz et al. (2015). Figure 3 summarizes results of these studies for all 23 colicins. We found that colicin activity and host range segregated into several groups. Some colicins showed relatively narrow specificity (e.g., active against only 1–2 strains); some demonstrated a moderately broader activity spectrum (e.g., active against 3–4 strains); and very few colicins (i.e., only ColM, ColIa, and ColIb) exhibited a broad activity spectrum. Based on our data, colicin cocktails composed of several colicins with complementary activity spectra (e.g., 2-component or 4-component blends such as ColM + ColIb + ColU + ColK) should be capable of controlling most pathogenic EHEC strains.

We developed two types of downstream purification processes to isolate colicins from plant biomass (Schulz et al., 2015). Extraction of the biomass followed by ultra/diafiltration and concentration results in COLICIN CONCENTRATE with typically 40–50% product purity. This approach is intended for use when edible plant species are used as expression hosts, because the components of the biomass are food and hence recognized as safe. If *N. benthamiana* is used as the production host, the downstream process includes a chromatography step resulting in COLICIN ISOLATE with higher purity.

A simple purification protocol comprizing extraction, ion exchange chromatography and dialysis resulted in 71% (ColK) – 97% (ColM) protein purity (Stephan et al., 2017). In the case of protein purity below 95%, detected protein impurities were found to be colicin degradation products. Supplementary Figure S1 summarizes purification data for three non-consecutive batches of ColM with an average 97.65% protein purity and 67.71% recovery. This protocol also provides for the efficient elimination of plant alkaloids down to safe levels: 22–171 ng/mg protein for nicotine and between undetectable levels and 44 ng/mg for anabasine (Stephan et al., 2017).

Purified colicin proteins were used for stability studies. We compared antimicrobial activities of ColM, ColU, ColIb, and ColK upon storage as solutions and as lyophilized powders at 4°C and room temperature for up to 309 days (ColK), 447 days (ColM and ColIb), and 552 days (ColU) (Supplementary Figure S2). All four lyophilized colicins retained their antimicrobial activities during the entire storage period at both 4°C and room temperature. Colicin M, Ib, and U demonstrated high stability also in solution when stored at 4°C (Supplementary Figure S2). These three colicins were least stable in solution at room temperature, with retention of activities under such conditions for 2 weeks (ColIb), 3 weeks (ColU), and 8 weeks (ColM). ColK solution was the least stable, with activity significantly declining after 1 week of storage either at room temperature or at 4°C (Supplementary Figure S2B). These data suggest that colicin preparations should be preferably stored in a dry form and reconstituted with water shortly before use. Ideally, colicin solutions should be refrigerated and used within a few weeks of preparation depending on the colicin cocktail composition.

**FIGURE 4:**
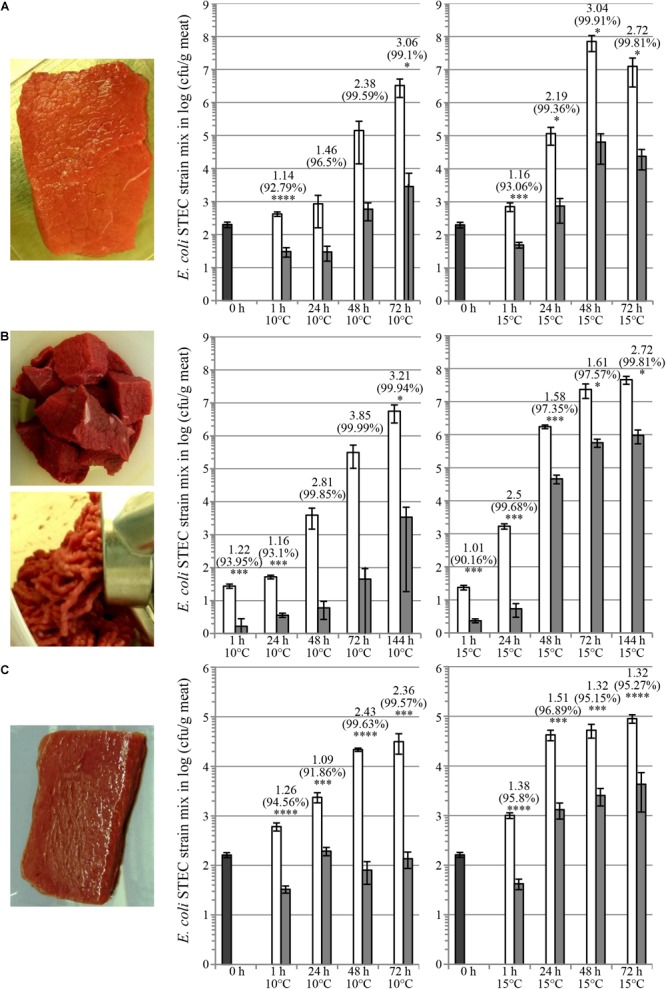
Reduction of shiga-toxin producing *E. coli* on fresh meat matrices. Fresh raw meat trims as beef steak **(A)**, beef meat cubes (before grinding) **(B)** and lamb loin filet **(C)** as shown in images were contaminated with a bacterial strain mix in equal cell number proportions of Big7 and O104:H4 serotypes (nalidixic acid resistant derivatives of strains CDC 03-3014, CDC 00-3039, CDC 06-3008, CDC 2010C-3114, CDC 02-3211, CDC 99-3311, ATCC^®^ 35150^TM^, and ATCC^®^ BAA-2326^TM^) either by dipping of steaks into bacterial solution **(A,C)** or by intermixing bacterial solution with beef cubes **(B)**. Subsequently, contaminated meat was treated with TSP extracts containing colicins by spraying at an application rate of 3 + 1 + 1 + 1 + 1 + 1 mg/kg colicins M + E7 + Ia + 5 + K + U. Beef cubes were used to prepare ground beef upon colicin treatment **(B)**. Graphs show bacterial populations recovered from meat on SMAC medium supplemented with 25 μg/ml nalidixic acid upon storage for various periods of time at 10 or 15°C upon colicin treatment (dark gray bars, initial contamination level; white bars, carrier treatment; light gray bars, bacteriocin treatment). Error bars indicate standard deviation of biological replicates, *N* = 4. Data annotations above bars correspond to mean log_10_ (cfu/g) reduction carrier vs. colicin treatment (upper line), mean percent (cfu/g) reduction carrier vs. colicin treatment (middle line) and statistical analysis by unpaired parametric *t-test* with GraphPad Prism v. 6.01 comparing the treatments at one timepoint with significance levels indicated by asterisks [^∗^*p* < 0.05 (probability of error less than 5%); ^∗∗^*p* < 0.01 (probability of error less than 1%); ^∗∗∗^*p* < 0.001 (probability of error less than 0,1%); ^∗∗∗∗^*p* < 0.0001 (probability of error less than 0,01%)].

Cocktails of plant-made colicins have been tested for control of EHEC contamination on various food matrices, including pork filet, beef steak, beef meat cubes (before grinding), and lamb loin filet. Previously, we reported the reduction of bacterial contamination of *E. coli* O157:H7 on fresh pork meat by treatment with colM + ColE7 mix (Schulz et al., 2015). Figure 4 shows decontamination of beef steak (A), beef meat cubes (before grinding) (B), and lamb loin filet (C). In these studies meat matrices were contaminated with a mixture of USDA “Big7 STEC” plus O104:H4 serotypes (8 strains in total). Colicin cocktail (M + E7 + Ia + 5 + K + U) treatment provided 1–3 logs reduction of bacterial population.

**FIGURE 5:**
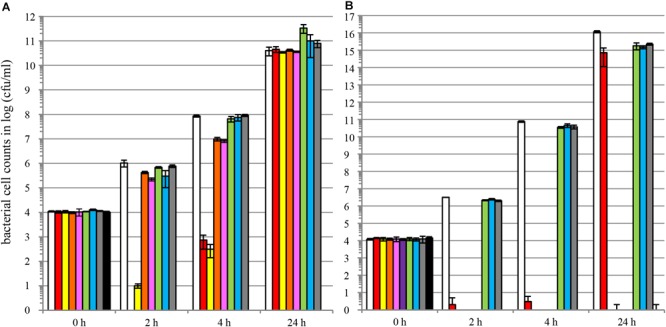
Colicins are able to control MDR-resistant shiga-toxin producing *E. coli* and colicin cocktails prevent development of colicin-insensitivity. LB liquid broth cultures of *E. coli* strain ATCC^®^ BAA-2326^TM∗^ of serotype O104:H4 were supplemented with different doses of individual colicins **(A)**, colicins blends **(B)**, antibiotics of different classes **(A,B)** or carrier [buffer solution, **(A,B)**]. The graphs show bacterial cell numbers upon co-incubation with antimicrobial test solutions [**(A,B)** white bars □, carrier; green bars 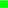, carbenicillin 50 mg/L; blue bars 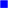, streptomycine 50 mg/L; gray bars 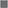, tetracycline 50 mg/L; black bars ■, kanamycin 50 mg/L; red bars 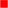, **(A)** colM 0.5 mg/L or **(B)** colM 5 mg/L; yellow bars 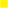 **(A)** colE7 0.5 mg/L or **(B)** colM + colE7 4.5 + 0.5 mg/L; orange bars 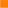, **(A)** colK 0.5 mg/L or **(B)** colM + colE7 + colE2 + colE6 3.5 + 0.5 + 0.5 + 0.5 mg/L; pink bars 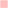 **(A)** colIa 0.5 mg/L or **(B)** colM + colE7 + colE2 + colE6 + colK + col5 2.5 + 0.5 + 0.5 + 0.5 + 0.5 + 0.5 mg/L; lilac bars 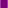 **(B)** colM + colE7 + colE2 + colE6 + colK + col5 + colIa 2.0 + 0.5 + 0.5 + 0.5 + 0.5 + 0.5 + 0.5 mg/L] for different timepoints at 37°C. Bacterial cell numbers were quantified by dilution plating (average of *N* = 3 samples, error bars correspond to SD) at timepoints 0, 2, 4, or 24 h of incubation. Experiments were performed using colicin-containing TSP extracts.

We also demonstrated that colicins are able to control multi-drug resistant *E. coli*. Figure 5 compares antimicrobial activities of colicins and antibiotics against MDR *E. coli* strain ATCC^®^ BAA-2326^TM^ of serotype O104:H4. This strain is positive for virulence genes *aggR* and *stx2* and negative for virulence genes *stx1* and *eae*. Genome sequencing revealed the presence of acquired antibiotic resistance genes, including β-lactamase of TEM-1 type, β-lactamase of CTX-M-15 type, multidrug-resistance gene cluster (*dfA7, sul1, sul2, strA, strB, tetA*, mercury resistance) and the tellurite resistance gene cluster (Rohde et al., 2011). ATCC^®^ BAA-2326^TM^ is resistant to ampicillin, piperacillin, cefazolin, cefotaxime, ceftazidime, cefepime, and trimethoprim/sulfamethoxazole. The strain is sensitive to cefoxitin, ertapenem, imipenem, amikacin, gentamicin, tobramycin, ciprofloxacin, levofloxacin, tigecycline, and nitrofurantoin^5^.

We compared individual colicins (Figure 5A) and colicin blends (Figure 5B) to four antibiotics representing three structural classes and two modes of action: carbenicillin (β-lactams, inhibitor of cell wall biosynthesis), streptomycin and kanamycin (aminoglycosides, inhibitors of protein biosynthesis), and tetracycline (tetracyclines, inhibitors of protein biosynthesis). We evaluated colM, colE7, colK, and colIa individually as well as in several blends. As expected, carbenicillin, streptomycin and tetracycline did not influence growth of the *E. coli* strain tested, whereas kanamycin eradicated bacterial cells. Individual colicins M and E7 significantly decreased bacterial population during the first 4 h of cultivation (Figure 5A). Colicin blends were much more efficient than individual colicins; colicin mixes M + E7, M + E7 + E2 + E6 and M + E7 + E2 + E6 + K + 5 completely eradicated bacterial cells (Figure 5B). The colicin effect was shown to be dose dependent; for example, colicin M used alone provided much more stringent bacterial control at 5 mg/l concentration compared to 0.5 mg/l.

### Plant-Made Salmocins

In contrast to the well-studied *E. coli* colicins, prior to our report (Schneider et al., 2018) colicin-like bacteriocins from *Salmonella* were scarcely studied (Patankar and Joshi, 1985). Based on homology to colicins, we identified in GenBank^®^ five *Salmonella* sequences coding for bacteriocins that we termed salmocins (*Salmonella* colicins): SalE1a and SalE1b with pore-forming activity and SalE2, SalE3, and SalE7 with nuclease activity. We successfully expressed all these proteins in *N. benthamiana* plants using the magnICON^®^ system at levels of 1.0–1.7 mg/g FW (Schneider et al., 2018).

Surprisingly, screening for antimicrobial activity against *S. enterica* ssp. *enterica* revealed unusually broad specificity and high activity for salmocins SalE1a and SalE1b (Schneider et al., 2018). These bacteriocins were active against all 109 test strains representing 105 pathogenic serovars with specific activities between 2 and 8 logs of AU/μg protein. Nuclease salmocins SalE2, SalE3, and SalE7 had narrower specificity (Schneider et al., 2018).

**FIGURE 6:**
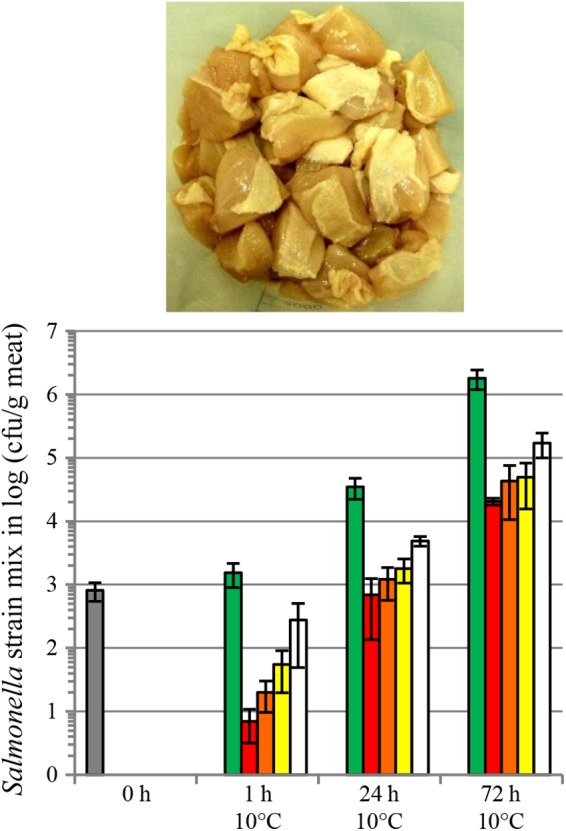
Reduction of a *S. enterica* ssp. *enterica* contamination on fresh skin-on chicken breast filet by salmocins. The graph shows bacterial populations recovered from meat shown in the picture upon storage for various periods of time at 10°C upon salmocin treatment (gray bar, initial contamination level; green bars, carrier treatment; red bars, salmocin treatment SalE1b in concentration of 5 mg/kg meat; orange bars, SalE1b in concentration of 1 mg/kg meat; yellow bars, SalE1b in concentration of 0.5 mg/kg meat; white bars, SalE1b in concentration of 0.1 mg/kg meat) of contaminated meat by spray-application. Error bars indicate standard deviation of biological replicates, *N* = 4. Statistically significant reductions (*p* < 0.005) in bacterial contamination were found by assessment of viable bacterial counts obtained from salmocin-treated in relation to carrier-treated meat samples by analysis by unpaired parametric *t-test* with GraphPad Prism v. 6.01 at all timepoints showing efficacy of salmocin treatment. Experiments were performed analogously to Schneider et al. (2018) (Figure 4) on meat contaminated with nalidixic acid resistant mutants of *Salmonella* strains of seven serovars mixed in equal cell number proportions [Enteritidis (ATCC^®^13076^TM∗^), Typhimurium (ATCC^®^14028^TM∗^), Newport (ATCC^®^6962^TM∗^), Javiana (ATC1^®^C0721^TM∗^), Heidelberg (ATCC^®^8326^TM∗^), Infantis (ATCC^®^BAA-1675^TM∗^), Muenchen (ATCC^®^8388^TM∗^)] and using semi-purified salmocin SalE1b protein. The purity of salmocin E1b was determined by capillary gel electrophoresis as described in Stephan et al. (2017) and found to be about 55% of total purified protein.

**FIGURE 7:**
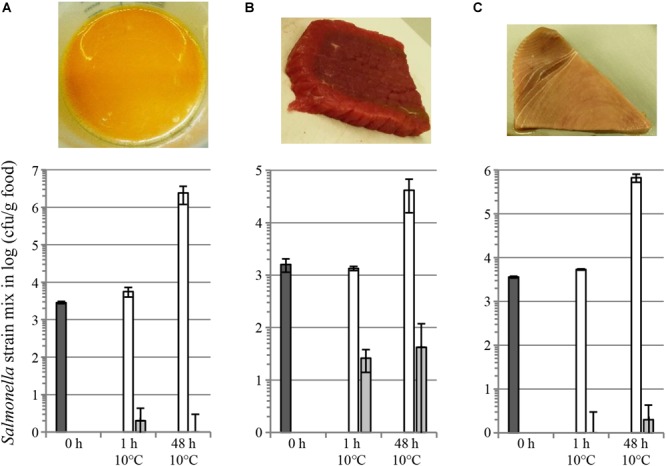
Reduction of a *S. enterica* ssp. *enterica* contamination on different food matrices by salmocins. Fresh raw food matrices as whole egg **(A)**, beef trims **(B)** and tuna filet trims **(C)** as shown in images were contaminated with a bacterial strain mix in equal cell number proportions [nalidixic acid resistant mutants of *Salmonella* strains of two serovars: Enteritidis (ATCC^®^13076^TM∗^) and Typhimurium (ATCC^®^14028^TM∗^)]. The graphs show bacterial populations recovered from foods shown in the pictures above upon storage for various periods of time at 10°C upon salmocin treatment (dark gray bars, initial contamination level; white bars, carrier treatment; light gray bars, salmocin treatment with SalE1b in concentration of 0.5 mg/kg food of contaminated food by intermixing. Error bars indicate standard deviation of biological replicates, *N* = 4. Statistically significant reductions (*p* < 0.005) in bacterial contamination were found by assessment of viable bacterial counts obtained from salmocin-treated in relation to carrier-treated food samples by analysis by unpaired parametric *t-test* with GraphPad Prism v. 6.01 at all timepoints showing efficacy of salmocin treatment. Experiments were performed using semi-purified salmocin SalE1b protein.

We also evaluated salmocins as antibacterials for *Salmonella* on several food matrices, including skinless chicken meat, skin-on chicken meat, beef steak, tuna filet and raw whole eggs. Food products were spiked with a mixture of seven *S. enterica* ssp. *enterica* strains representing seven (Enteritidis, Typhimurium, Newport, Javiana, Heidelberg, Infantis and Muenchen) or two (Enteritidis, Typhimurium) pathogenic serovars in the case of chicken or other food matrices, respectively. Efficient decontamination of skinless chicken meat with individual salmocin SalE1a and salmocin blend SalE1a + SalE1b + SalE2 + SalE7 was described in Schneider et al. (2018). Figure 6 shows a significant (1–2 log) reduction of a *Salmonella* contamination on fresh skin-on chicken breast filet by individual salmocin E1b used in several concentrations: 5.0, 1.0, 0.5, and 0.1 mg/kg meat. Figure 7 shows reduction of *Salmonella* contamination on whole egg (A), beef trims (B) and tuna filet trims (C) by SalE1b. SalE1b in a concentration of 0.5 mg/kg food provided bacterial load reduction of 3–8 logs in whole egg, 1.8–3 logs in beef trims, and 3.8–5 logs in tuna filet.

**FIGURE 8:**
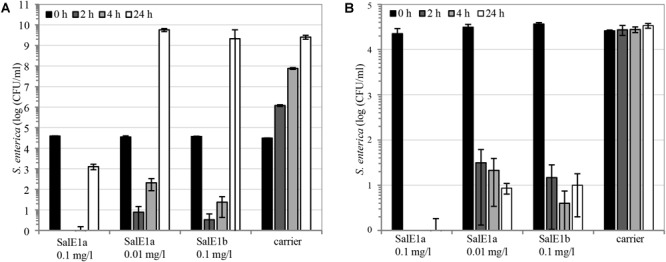
Antibacterial activity of salmocins on *S. enterica*. LB liquid broth cultures consisting of a mixture of *S. enterica* ssp. *enterica* serovars Enteritidis and Typhimurium (strains ATCC^®^ 13076^TM∗^, and ATCC^®^ 14028^TM∗^, respectively) were supplemented with indicated doses of individual salmocins or carrier (buffer solution). Cultures were incubated **(A)** at 37°C or **(B)** at 10°C and the reduction of bacterial cell numbers was quantified by dilution plating (average of *N* = 3 samples, error bars correspond to SD) at timepoints 0, 2, 4, or 24 h of incubation. Experiments were performed using salmocin-containing TSP extracts.

We searched for the lowest industrially practical application rates for salmocins to control *Salmonella*. *In vitro*, pore-forming SalE1a and SalE1b were highly active against the mix of two *Salmonella* strains of representative serotypes Enteritidis and Typhimurium at low concentrations of 0.1 and 0.01 mg/l (Figure 8). Interestingly, low temperature (10°C, Figure 8B) did not have a significant impact on these salmocins’ bactericidal effect compared to their activity at 37°C (Figure 8A).

We also compared four types of antimicrobial proteins of different origin (colicins and salmocins from Gram-negative bacteria, *Listeria* phage endolysins from pathogens of Gram-positive species, and nisin from Gram-positive species) for their activity against Gram-negative *E. coli* and *S. enterica*, and Gram-positive *L. monocytogenes* (Supplementary Figure S3). Nisin, a food-approved bacteriocin which is widely used commercially, is a low molecular weight peptide originating from the Gram-positive bacterium *Lactococcus lactis*. Plant extracts containing corresponding proteins were tested against mixes of bacterial strains listed in Supplementary Figure S3B. We found *E. coli* to be sensitive to colicins only (Supplementary Figure S3A). *Salmonella* was most sensitive to salmocins and, to a lesser extent, to colicins. Interestingly, *Salmonella* also showed little sensitivity to the mix of *Listeria* phage endolysins (Supplementary Figure S3A). *Listeria* was completely insensitive to both colicins and salmocins; it showed only slight sensitivity to endolysins but high sensitivity to nisin (Supplementary Figure S3A). Our data indicate a clear distinction in specificities between antimicrobial proteins derived from Gram-positive and Gram-negative bacterial species without significant cross-activity between these two classes of microorganisms.

### Production of Colicins and Salmocins in Ethanol-Inducible Transgenic Plant Hosts

The large scale manufacture of antimicrobial proteins for food use will require processing large amounts of plant biomass and low production cost. We believe that ethanol-inducible protein expression using a transgenic plant host is more amenable to cost-efficient scale-up than a transient expression approach. We already reported on the development of ethanol-inducible transgenic *N. benthamiana* lines for the expression of colicin M (Schulz et al., 2015) and salmocin E1b (Schneider et al., 2018). Currently, we are developing transgenic *N. benthamiana* lines for production of other colicins and salmocins. Alternative approaches for large-scale protein expression in plants, such as agroinfiltration or agrospray, require a fermentation facility to generate inoculum, plus containerization, plant transport and vacuum infiltration equipment in infiltration-based processes (Chen et al., 2013; Gleba et al., 2014; Tusé et al., 2014). Such process requirements introduce complexity and ultimately drive up manufacturing capital and operating costs. Although higher cost of goods sold (cogs) might be tolerated for pharmaceutical or other high-value products produced through transient expression, they are undesirable in cost-constrained applications such as food safety.

**FIGURE 9:**
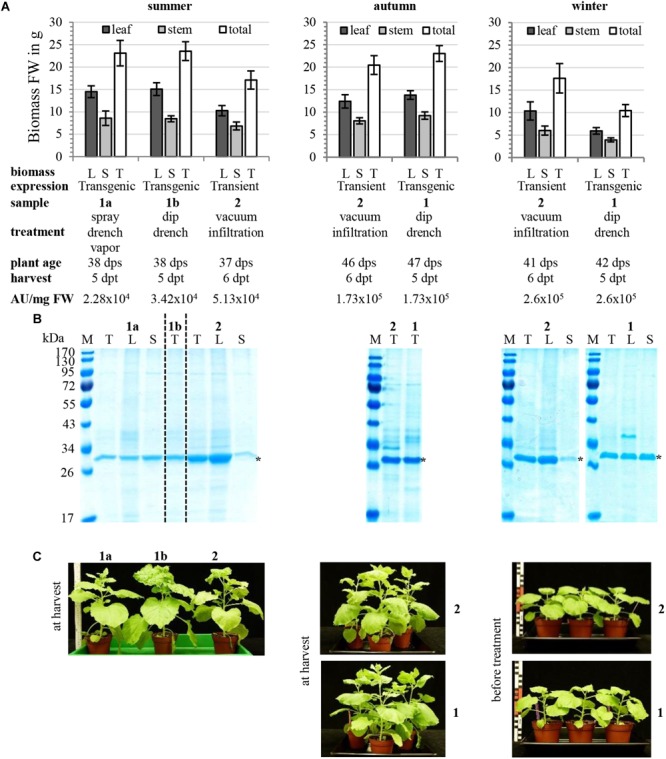
Transgenic EtOH-inducible hosts for colicin expression. Independent experiments performed in different seasons, summer, autumn, and winter, comparing transient and transgenic colicin M expression are shown. Methods of plant cultivation, transient expression of colicin M using *N. benthamiana* WT plants and vacuum infiltration of 1:100 dilutions of agrobacterial cultures or ethanol-induction of transgenic plants using 4% (v/v) EtOH solutions were described in Werner et al. (2011) or Schulz et al. (2015), respectively. The TMV-based constructs for transient colM expression (pNMD10221) and for EtOH-inducible colM expression (pNMD18381) were described in Schulz et al. (2015), Supplementary Figures S1, S3, respectively. Transgenic plants for EtOH-inducible colM expression used were T4 generation plants homozygous for single copy T-DNA insertion with characterized T-DNA ends and genomic insertion point of *N. benthamiana* plant line Nb18381T0#29 initiated as described in Schulz et al. (2015) (Supplementary Figure S2). **(A)** shows the yield of plant biomass in g fresh weight (FW) as average and standard deviation of 6 or 3 plants (also true for **B**) and sample description giving plant age at timepoint of treatment (vacuum infiltration or EtOH-induction) in days post sowing (dps), harvesting timepoint for biomass and recombinant protein analysis in days post treatment (dpt) and antimicrobial activity of colicin-containing TSP extracts in AU/mg FW plant biomass. **(B)** inspection of plant TSP extracts prepared with 2 vol. 20 mM citrate, 20 mM Na_2_HPO_4_, 30 mM NaCl, pH 4.0 using Angel Juicer 7500 and feeding buffer using peristaltic pump iPump2S by SDS-PAGE and Coomassie-staining; recombinant colM is marked with asterisks, loading corresponds to 2.5 mg FW (summer and winter) or 3.75 mg FW (autumn). **(C)** Plant phenotypes before treatment or at harvest.

Here we analyzed the performance of colM-producing *N. benthamiana* line (T4 generation plants homozygous for single-copy T-DNA insertion) depending on season; we also compared transient and transgenic colicin M expression (Figure 9). In our semi-controlled glass-facade greenhouse conditions, seasonal differences in plant biomass yield depend mostly on light intensity and the lowest amount of plant biomass was found in the winter season due to slower plant growth as this is usually observed for all plant species grown in the greenhouse (Figure 9A). The lower biomass yield of seasons with unfavorable speed of plant growth can be compensated by prolonged incubation of plants before treatment and harvest, as seen in comparison of summer and autumn plants (Figure 9A). There was no prominent difference between transgenic and transient production host (Figure 9A). Expression of recombinant proteins was more equally distributed between leaves and stems for transgenics compared to vacuum-infiltrated plants with predominant leaf expression (Figure 9B). Despite experiment-to-experiment variability, transgenic and transient expression hosts provided comparable levels of recombinant protein accumulation (Figure 9B). Phenotypically, transgenic plants were indistinguishable from wild type plants (Figure 9C).

### Colicins/Salmocins: Regulatory Marketing Allowance as Food Antimicrobials

Development and regulatory approval of any product to be added to food or used as medicine is a complex, lengthy and usually costly process. Regulatory approvals for food additives vary significantly from country to country. We discuss here regulatory approval pathways for food antibacterials in the United States because this country represents by far the largest potential market for these products and because its regulatory review process can be relatively simple and fast (and relatively inexpensive), compared to the regulations in most other countries. In the United States, any substance to be intentionally added to food is a food additive and must be subjected to premarket review and approval by the FDA under the Federal Food, Drug and Cosmetic Act (FFDCA; the “Act”), unless the substance meets a listed exemption in the Act, or is generally recognized, among qualified experts, as having been adequately shown to be safe under the conditions of its intended use^6^.

GRAS’ (Generally Recognized As Safe) is an FDA designation that a chemical or substance added to food is considered safe by experts, and so is exempted from the conventional premarket approval process by FDA. The developer of the new substance (the Notifier) conducts an analysis of safety and utility of its product using scientific procedures including corroboration from publically available information, and determines and documents that the substance is GRAS as specified by the FDA’s Final Rule for GRAS Notices^7^. The Notifier voluntarily submits its GRAS conclusion to FDA for review and comment. The FDA can either reject Notifier’s conclusion of safety, cease to evaluate the submission upon request by the Notifier, or, ideally, issue a “No Questions” letter to Notifier. The latter verifies that the FDA agrees with Notifier on its conclusion that the substance is GRAS and equates to marketing allowance by FDA for the substance. The FDA may conduct the GRAS review on its own for certain types of food treatments, or solicit input from the United States Department of Agriculture (USDA) if the substance is to be applied to USDA-regulated products such as meat and egg products. The GRAS pathway can be used for substances added to human food or animal food, as well as for animal feed ingredients.

In addition to the FDA, an alternative body capable of conducting GRAS reviews is the Flavor and Extract Manufacturers Association (FEMA), which is the national association of the United States flavor industry. FEMA works with legislators and regulators to assure that the needs of members and consumers are addressed and can provide GRAS guidance, although their function is restricted to flavor substances.

The GRAS designations fall into several categories, the most relevant of which for food antibacterials is “Food Processing Aid.” Substances added to food are classified as Food Processing Aids if they provide a rapid yet temporary effect, degrade and become part of the food matrix and thus have no functional effect on the food. If FDA accepts such a designation based on the evidence provided, the designation allows the manufacturer to avoid listing the substance on the treated food’s product label; thus, there is no labeling requirement for the substance. Food additives or food ingredients, on the other hand, are typically persistent, are essential to or can modify the food’s functionality, and need to be listed on the product label.

Facilitated regulatory pathways similar to GRAS exist also in a few other countries, for example Canada, Mexico, Australia, and New Zealand. In yet other territories, including countries of the European Union and Japan, approval of a new food additive involves a process similar to the USA’s pre-market review of a new non-GRAS substance, requiring extensive toxicity/safety studies.

Nomad Bioscience is the first company to successfully obtain FDA concurrence for GRAS designation of its plant-made bacteriocins, such as colicins, as food antimicrobials. In its first GRAS notice (GRN 593^8^), the following arguments were used to support safety and suitability of colicins made in food species hosts as food antimicrobials.

Safety:

– Colicins are naturally occurring antibacterial proteins produced endogenously by commensal enteric bacteria in the human gut;
– There is an extensive documented history of colicin exposure in humans and other animals;
– Colicin exposure occurs naturally from commensal bacteria as well as from food;
– There is no documented etiologic relationship between colicins and disease;
– Colicins are notoriously unstable to heat, acid and proteolytic digestion;
– There are no reports of colicins being allergenic or posing a hypersensitivity risk;
– The manufacturing process described utilizes food plants to produce recombinant colicins;
– The compositions of the plant-made recombinant colicin proteins match those of the bacterial colicin proteins; and
– The recombinant plant-made colicins are not glycosylated, just as the native bacterial colicins.

Suitability:

– The target specificities of plant-made colicins match those reported for the bacterial colicins;
– The specific activities (potency) of plant-made colicins are in the range of those reported for bacterial colicins;
– Colicins (plant-made as well as native bacterial) exhibit complementary and synergistic activities and can be used as mixtures depending on the intended use and target pathogen(s);
– Nomad’s product, COLICIN, is comprised of single colicins or mixtures thereof;
– COLICIN can be formulated at different purities depending on the intended end use;
– COLICIN is active against target food pathogens at use rates not to exceed 10 mg COLICIN/kg food;
– COLICIN is bactericidal on fruits and vegetables, and it can be used to treat bulk raw produce (wash), processed/cut produce (wash, dip, or spray), or included as a package additive in ready-to-eat fruits and vegetables.

In 2015, based on Nomad’s submission, FDA accepted colicins as first-in-class GRAS antimicrobials for controlling pathogenic *E. coli* in fruits and vegetables (GRN 593), and in 2017, FDA and USDA accepted colicins as antimicrobials for controlling *E. coli* in meat products (GRN 676^9^). In both cases, the Agencies agreed with the “Food Processing Aid” definition and USDA has added colicins to its FSIS Directive 7120, which is a list of safe and suitable ingredients allowed for use in the production of meat, poultry and egg products (FSIS Directive 7120.1, Revision 42). Subsequently, Nomad also filed GRAS notices for *Salmonella* salmocins (GRN number pending) and *C. perfringens* bacteriophage endolysins (GRN 802^10^). Independently, Nomad also submitted a GRAS notice for the use of *N. benthamiana* (non-edible) production host for the manufacture of colicins (GRN 775^11^), which led to a “No Questions” letter from FDA. A list of allowed GRAS notices, notices currently under review by FDA, and notices in preparation is provided in Supplementary Table S1.

Regulatory experience to date suggests that additional plant-made colicins, phage-derived endolysins, other bacteriocins, defensins, etc., could also gain rapid marketing allowance. In particular, in 2018 Nomad received ‘No Questions’ letter from FDA for a product candidate in another category of food treatments, the natural sweeteners/taste modifiers thaumatins (GRN 738^12^). As long as the GRAS notice describes a natural or nature-identical substance, GRAS designation is a relatively simple, fast and inexpensive way for obtaining regulatory review and product marketing allowance, as evidenced by the success of Nomad’s GRAS submissions to date. Based on this experience, we see a potential GRAS allowance ‘space’ for multiple classes of natural proteins such as:

– Colicins/bacteriocins-based feed treatments as antibiotic alternatives for controlling bacteria in animals during life or prior to harvesting;
– Antivirals such as antibodies or lectins (griffithsin) added to food (e.g., control of norovirus, rotavirus, influenza);
– Functional (medicinal) foods to control bacteria or viruses in the gastrointestinal tract;
– Bacteriocins as topical/oral treatments;
– Natural proteins (e.g., thaumatin) as non-caloric sweeteners, taste modifiers, etc.

Natural products that are used to treat food but that have no functional effect on food (food processing aids) are obviously the easiest cases to take through the GRAS process. For colicins, the inherent safety of these proteins was supported in part by the fact that colicins and colicin-like bacteriocins are very sensitive to proteases, and any traces of these proteins remaining in the treated food would be rapidly degraded in the stomach and duodenum; Nomad provided FDA with extensive data on gastroduodenal degradation of colicins in its dossier. Future uses of bacteriocins as food treatments to control bacteria in the human or animal gastrointestinal tract would likely require additional data on bacteriocin safety, bioavailability, and their functionality in the intestinal lumen.

### Potential Markets for Bacteriocins as Food Antibacterials

Bacteriocins such as colicins and salmocins are promising alternatives to antibiotics for many markets, perhaps most importantly for food and medicinal uses. At present, antibiotics still constitute our main therapeutic toolbox for controlling pathogens; the situation is, however, rapidly changing with increasing number of pathogenic bacteria becoming resistant to most antibiotics. In the health care market, bacteriocins could probably be effectively used today in specific market niches in which the pathogen species are known and a topical application (direct surface delivery to skin, surface of lungs, surface of urogenital tract, and intestinal tract) is possible. Such indications could include treatment of cystic fibrosis patients by inhalation-based delivery of pyocins to treat *Pseudomonas*, or treatment of uropathogenic *Escherichia* by catheter delivery of colicins (Tables 1, 2).

**Table 2:** Potential uses of bacteriocins in food and medicines/medical devices.

Food antimicrobials	Medicines/medical devices
Limited but growing market, unmet needs	Almost unlimited market, unmet needs
Antibiotics not acceptable	Antibiotics used but loosing efficacy because of multi-drug resistance
Fast approval in United States, Canada, few other countries, slow and expensive approval in the rest of the world	Fast approval world-wide as medical devices, expensive approval as medicines
Low development cost (United States)	Lower development cost as medical device, higher development cost as medicine
Natural molecules only (for fast approval)	Engineered molecules acceptable
No strategic partners or history of product development	Multiple strategic partners during late R&D phases but overall low interest in antibiotic market
Plants produce natural bacteriocins efficiently	Plants produce natural and engineered bacteriocins efficiently
Clearly defined pathogens as target for product candidates	Clearly defined pathogens as target for product candidates
Multiple GRAS acceptances/filings	One medical device approved, two Phase I trials

Potential markets for food antibacterials are large and even more immediate because of the facile marketing channels in countries such as United States and Canada. Table 3 lists potential market segments that are in need of better food safety through bacterial control, and those markets are sizeable and numerous. Given the current antibacterial intervention costs accepted or acceptable by the industry ($0.025–0.1 per kg of food product), the estimated markets for antibacterials could potentially be very attractive. Some of the segments, such as processing of fresh and ground beef or processing of poultry and pigs, are oligopolistic with only a few companies controlling the majority of the United States market (i.e., Tyson Foods, JBS, Cargill, National Beef, and Pilgrim’s Chicken). Therefore, a commercial alliance with just one such partner would provide access to 20% or more of the market segment.

**Table 3:** Potential United States food/feed safety and animal health markets for bacteriocins.

Market segment	Intervention price, $/kg or per animal	Estimated market for antibacterials, $ M
Ground beef^∗^	0.025–0.1	47–350
Fresh beef^∗^	0.025–0.1	88–353
Ready-to-eat meats	0.025–0.1	75–153
Chicken, turkey, pigs^∗∗^	0.025–0.1	180–720
Pre-harvest poultry	0.025–0.1	1000
Animal health, mastitis	65 per cow	180
Post-weaning diarrhea	0.65 per piglet	50–100
Enteric diseases, poultry	0.025–0.05 per bird	170
Vegetables and lettuce^∗^	0.1	256
Bagged salads^∗^	0.125	100

*^∗^GRAS status agreement by FDA obtained; ^∗∗^GRAS status agreement by FDA expected Q1 2019*.

## Discussion

Food safety market needs are shaped by two major trends, both of which revolve around real and perceived food safety issues. The first trend is the rapid increase of multi-drug resistant forms among common bacterial pathogens present in food. The second is the desire by consumers to have a ‘natural’ food that is devoid of chemical additives or genetically modified ingredients (‘organic food’). This second trend is actively exploited by food companies because it allows them to charge a premium for food classified as ‘natural,’ ‘GMO-free,’ ‘antibiotic-free,’ ‘organic,’ ‘bio,’ etc. Unfortunately, the modification of agricultural and husbandry practices and subsequent food processing so as to exclude previously accepted chemical interventions drastically increases the likelihood of food contamination by bacteria. Two examples illustrate this point. Contamination of food with pathogenic *E. coli* was originally dubbed a ‘hamburger disease’ because such contamination was initially traced to contaminated beef. The statistics from the United States Centers of Disease Control and Prevention (CDC) that tracks outbreaks in United States demonstrate that during 2006–2010 only one out of ten outbreaks was traced to contaminated vegetables. However, during 2011–2016, nine out of thirteen *E. coli* foodborne outbreaks was due to vegetables/vegetable products, including five due to organically grown vegetables and sprouts. Another sad illustration is the case with Chipotle, a United States restaurant chain that in 2013 declared itself as the one intending to provide its customers with natural, organic and GMO-free foods. Within approximately the next 18 months, there were four outbreaks due to contamination involving three different pathogens (*E. coli, Salmonella*, and norovirus). As a result, the company stock took a serious hit and its market value had fallen by almost 65% by the end of 2017, somewhat recovering during the first half of 2018. However, in August 2018 the company experienced its largest outbreak to date, this one due to *C. perfringens*. In the minds of the general public, there still appears to be no correlation between ‘organic’ farming/food and higher risk of bacterial contamination. Nevertheless, since bacteriocins are natural proteins identical to the ones made by our intestinal bacterial flora, we believe that bacteriocins are more likely to be accepted as novel food safety interventions by the industry, non-governmental and governmental organizations and ultimately by consumers.

There is a concern about increase in resistance to colicins and salmocins upon their use as food antimicrobials. Bacterial resistance to bacteriocins is well-known, it was described in numerous publications (e.g., Riley and Gordon, 1996; Feldgarden and Riley, 1998; Kirkup and Riley, 2004, etc.). We believe that the application of colicins/salmocins to food is unlikely to allow for selection of bacteriocin-resistant bacteria in the intestinal tract of humans, for three main reasons:

– Cooking colicin/salmocin-containing foods prior to ingestion will thermally inactivate bacteriocin proteins;
– Even without cooking/heating, ingested colicins/salmocins will be denatured by the low pH of the stomach; and
– Colicin-class proteins will be rapidly digested by proteases in the upper and mid gastrointestinal tract before they reach the colon.

There are other serious challenges for the developers of bacteriocins as food antibacterials. The food industry has traditionally been reluctant to make significant investments to develop new products unless market or other dynamics mandate it. In particular, antibiotics have been developed by pharmaceutical companies and afterwards adopted by the food industry. Development of the most recent antibacterial products, bacteriophages, has been pioneered and conducted almost entirely by small companies and academia. Most of our discussions with large companies active in food production and processing indicated that they would consider adopting a bacteriocin product if it were available on the market already, but not if it was a product candidate in late development, even if it had already been accepted by regulatory agencies. In other words, a bacteriocin developer may be expected to shoulder the majority of the costs and risks of not only development and regulatory review of the product but also of building manufacturing capacity and marketing resources. This is in stark contrast to the pharmaceutical industry, where there is a “division of labor” in which the R&D pipeline is serviced by multiple small and medium size companies each specializing in certain steps of product development, such as discovery, preclinical studies, or Phases I–II clinical studies. The risk is compounded by the fact that in case of eventual acquisition of the whole product pipeline and accompanying infrastructure by a food industry company (the preferred exit for a small developer), the developer is unlikely to receive multiples on the investment made that are comparable to the multiples enjoyed by developers in the pharmaceutical business (i.e., depending on the development phase at trade sale, average 3.7–4.8 multiples on investment can be realized).

Additional challenges stem from the regulatory requirements imposed on the food industry. Whereas there are strict ‘zero tolerance’ rules concerning contamination of food with *E. coli*, there are, for practical reasons, no such limits on contamination with *Salmonella* or other food pathogens. Correspondingly, large food producers’ only “incentives” are the costs of food recalls due to contamination and the damage to their product brands, and sometimes, to their share price.

Some optimism is offered by recent successes of companies developing plant-based meat analogs, such as Beyond Meat and Impossible Foods. The latter company includes recombinant (yeast) leghemoglobin in its ingredients as a flavor enhancer, with the argument that it is less environmentally impactful to produce the heme protein recombinantly by fermentation than to obtain it by natural extraction of legumes (soybeans) grown in vast acreages. This concept appeals to the overall consumer base targeted by the company, whose business model is to offer meat-replacing foods that taste like meat but do not lead to deforestation to raise grains for animal feed, thereby combating global warming. The recombinant heme has achieved GRAS status (GRN 737^13^), and the plant-based meats are gaining acceptance with consumers in spite of GM ingredients. There is a lesson to be learned in this example that could apply to consumer acceptance of natural proteins such as colicins, salmocins and others that are known to be safe and effective, can address a major worldwide safety issue, and can be manufactured at scale in plant-based systems that are sustainable and environmentally compatible. The future will be interesting indeed.

## Author Contributions

SH-L, ASt, AG, and YG designed the research. SH-L, ASt, SS, TS, and ASh performed the research. SH-L, ASt, SS, TS, DT, AG, and YG analyzed the data. SH-L, DT, AG, and YG wrote the manuscript.

## Conflict of Interest Statement

The authors declare that the research was conducted in the absence of any commercial or financial relationships that could be construed as a potential conflict of interest.

## Footnotes

1 www.washingtonpost.com/news/wonk/wp/2015/08/05/there-are-a-lot-more-cows-in-a-single-hamburger-than-you-realize/?noredirect=on&utm_term=.defc863946a4

2 https://www.cdc.gov/ecoli/2018/o157h7-11-18/index.html

3 www.cdc.gov/foodsafety/outbreaks/index.html

4 https://www.govinfo.gov/content/pkg/FR-2011-09-20/pdf/2011-24043.pdf

5 https://www.lgcstandards-atcc.org/support/faqs/6e2c1/ATCC%20BAA-2326%20antibiotic%20resistant.aspx?device=modal

6 www.fda.gov/Food/IngredientsPackagingLabeling/GRAS/

7 www.federalregister.gov/documents/2016/08/17/2016-19164/substances-generally-recognized-as-safe

8 https://www.accessdata.fda.gov/scripts/fdcc/index.cfm?set=GRASNotices&id=593

9 https://www.accessdata.fda.gov/scripts/fdcc/?set=GRASNotices&id=676&sort=GRN_No&order=DESC&startrow=1&type=basic&search=676

10 https://www.accessdata.fda.gov/scripts/fdcc/?set=GRASNotices&id=802

11 https://www.accessdata.fda.gov/scripts/fdcc/?set=GRASNotices&id=775

12 https://www.accessdata.fda.gov/scripts/fdcc/index.cfm?set=GRASNotices&id=738

13 https://www.accessdata.fda.gov/scripts/fdcc/index.cfm?set=GRASNotices&id=737
