# Supplementary material for: Colicins and Salmocins – New Classes of Plant-Made Non-antibiotic Food Antibacterials
Source: Front Plant Sci. 2019 Apr 9;10:437. doi: 10.3389/fpls.2019.00437 (PMC6465592; doi:10.3389/fpls.2019.00437)
Supplement: Supplementary file 1 [file Data_Sheet_1.pdf]

Supplementary Material

1 Supplementary Data

2 Supplementary Figures and Tables

2.1 Supplementary Figures

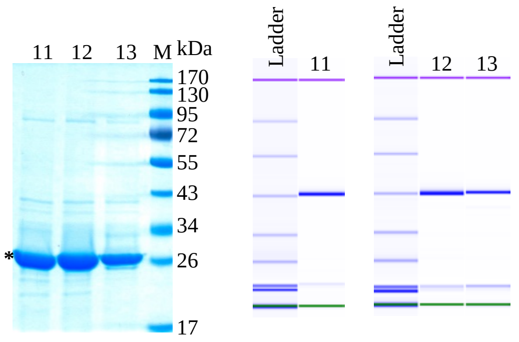

Purified colM Batch	Yield in mg/g FW	Purity in %	Recovery in %
11	1.56	97.05	58
12	1.05	97.75	79.7
13	1.39	98.15	50.4

Supplementary Figure S1. Small-scale purification of colicin M from *N. benthamiana*. Three non-consecutive batches (batch 11, 12 and 13) of purified colicin M were prepared and analyzed for purity by **(A)** SDS-PAGE or **(B)** CGE as described in Stephan et al., 2017.

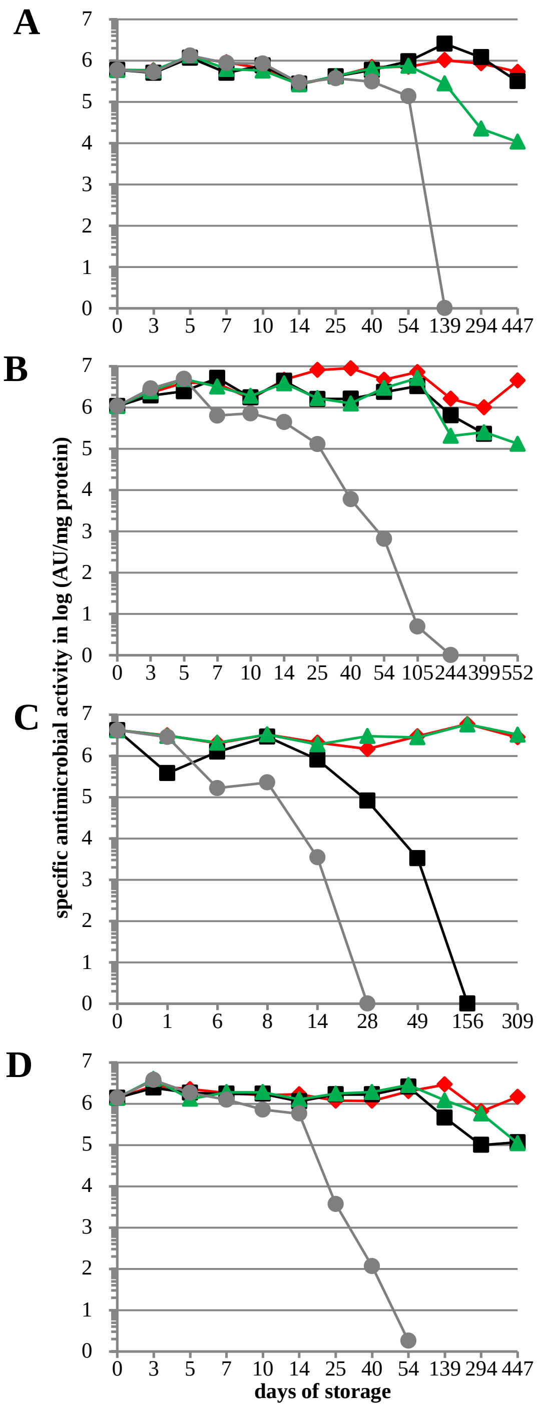

Supplementary Figure S2. Stability studies on purified colicins. (A) colicin M, **(B)** colicin K, **(C)** colicin U and **(D)** colicin Ib were purified as described in Stephan et al., 2017 and stored as lyophilized protein powders at 4°C (red lines) or at room temperature (green lines) or as solutions upon reconstitution of lyophilized protein powders with water at 4°C (black lines) or room temperature (grey lines). Stability of colicins was assayed by determination of the specific antimicrobial activity of colicins stored under different conditions in time course against *E. coli* DH10B test strain by softagar overlay assay as described in Schulz et al., 2015.

A

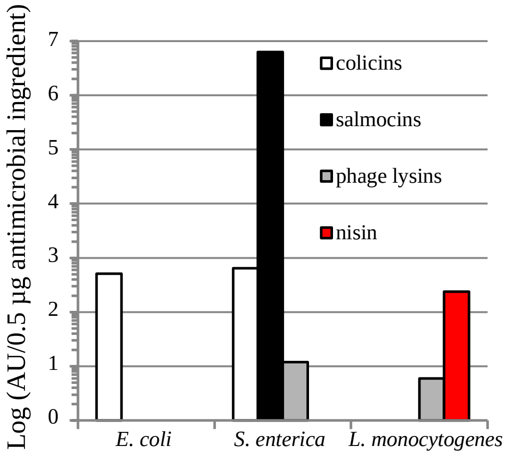

B

species	strain	serotype
Escherichia coli (STEC)	CDC 03-3014	O26:H11
	CDC 00-3039	O45:H2
	CDC 06-3008	O103:H11
	CDC 2010C-3114	O111:H8
	CDC 02-3211	O121:H19
	CDC 99-3311	O145:NM
	ATCC® 35150™	O157:H7
	ATCC® BAA-2326™	O104:H4
Salmonella enterica ssp. *enterica*	ATCC® 13076™*	Enteritidis
	ATCC® 14028™*	Typhimurium
	ATCC® 6962™*	Newport
	ATCC® 10721™*	Javiana
	ATCC® 8387™*	Montevideo
	ATCC®BAA-1675™	Infantis
	ATCC® 8388™*	Muenchen
	ATCC® 8326™*	Heidelberg
Listeria monocytogenes	WSLC 1685	1/2a, 4b
	WSLC 10207	1/2a
	WSLC 1001	1/2c
	WSLC 1020	4a
	WSLC 1042	4b
	WSLC 1019	4c
	WSLC 1033	4d
	WSLC 1018	4e

Supplementary Figure S3. Activity spectrum of antimicrobial proteins with different modes of action. Semi-quantitative evaluation of the antimicrobial activity of colicin-, salmocin- or phage lysin-containing plant TSP extracts was done by radial diffusion assay via spot-on-lawn-method as described in Schulz et al. 2015. Undiluted antimicrobial test solutions contained 0.1 mg/ml active ingredients of either a blend of 6 colicins (1:1:1:1:1:1 blend of colM, colE7, colK, colIa, col5 and colU), a blend of 5 salmocins (1:1:1:1:1 blend of salE1a (OIN35410.1), salE1b (OIN32443.1), salE2 (KTM78572.1), salE3 (GAS18013.1) and salE7 (KSU39545.1)), a blend of 8 *Listeria* phage lysins (1:1:1:1:1:1:1:1 blend of PlyA118 (CAA59362.1), PlyA500 (CAA59365.1), PlyA511 (CAA59368.1), PlyP35 (AAY53213.1), PlyP40 (HV559656.1), PlyPSA (CAC85577.1), PlyP70 (AFQ96195.1) and PlyA006 (YP_001468860.1)) or nisin (Nisin from *Lactococcus lactis* – 2.5% (balance sodium chloride and denatured milk solids), purchased from Sigma, #N5764)). **(A)** The specific antimicrobial activity was calculated in arbitrary units (AU) per recombinant protein (average of independent experiments, N=2, error bars correspond to SD). *E. coli* and *Salmonella* or *Listeria* test strains were cultivated using LB or Brain Heart Infusion medium, respectively. Bacterial test cultures were in each case a mixture of 8 individual strains in equal proportions, information of strains used is given in **(B)**. Of the species *E. coli*, the culture consisted of a strain mix of Big7 and O104:H4 serotype.

2.2 Supplementary Tables

Supplementary Table S1. Nomad’s GRAS notices and FDA acceptance

**Product/Origin**	**GRAS GRN**	**Submission date**	**Response date**
Colicins/*Escherichia coli*	**000593**	07.2015	**12.2015/FDA**
Colicins/*Escherichia coli*	**000676**	11.2016	05.2017/FDA<br>01.2017/USDA
Thaumatins/*Thaumatococcus*	**000738**	10.2017 (sweetener)<br>est. 12.2018<br>(taste modifier)	**04.2018/FDA**<br>est. 06.2019
Salmocins/*Salmonella enterica*	000824	11.2018	est. 05.2019
Endolysins/*Chlostridium difficile*	000802	07.2018	est. 01.2019
Colicins/*Escherichia coli*	**000775**	04.2018	**10.2018/FDA**
Colicins/food additives		06.2019	est. 12.2019
Salmocins/food additives		09.2019	est. 03.2020

Notices with confirmed FDA GRAS status are shown in **boldface**.
